# Design of the study: How can health care help female breast cancer patients reduce their stress symptoms? A randomized intervention study with stepped-care

**DOI:** 10.1186/1471-2407-12-167

**Published:** 2012-05-04

**Authors:** Karin Nordin, Ritva Rissanen, Johan Ahlgren, Gunilla Burell, Marie-Louise Fjällskog, Susanne Börjesson, Cecilia Arving

**Affiliations:** 1 Department of Public Health and Caring Sciences, Uppsala University, Box 564, Uppsala, SE-751 22, Sweden; 2 Department of Public Health and Primary Health Care, University of Bergen, Bergen, Norway; 3 Department of Oncology, Gävle hospital, and Centre for Research and Development, Uppsala University, Uppsala, Sweden; 4 Department of Oncology, Radiology and Clinical Immunology, Uppsala University, Uppsala, Sweden; 5 Department of Surgery, Central Hospital of Falun, Falun, Sweden

**Keywords:** Breast cancer, Randomized multicenter interventions study with stepped-care approach, Stress management

## Abstract

**Background:**

A life threatening illness such as breast cancer can lead to a secondary diagnosis of PTSD (post traumatic stress disorder) with intrusive thoughts and avoidance as major symptoms. In a former study by the research group, 80% of the patients with breast cancer reported a high level of stress symptoms close to the diagnosis, such as intrusive thoughts and avoidance behavior. These symptoms remained high throughout the study. The present paper presents the design of a randomized study evaluating the effectiveness and cost-effectiveness of a stress management intervention using a stepped-care design.

**Method:**

Female patients over the age of 18, with a recent diagnosis of breast cancer and scheduled for adjuvant treatment in the form of chemotherapy, radiation therapy and/or hormonal therapy are eligible and will consecutively be included in the study. The study is a prospective longitudinal intervention study with a stepped-care approach, where patients will be randomised to one of two interventions in the final stage of treatment. The first step is a low intensity stress-management intervention that is given to all patients. Patients who do not respond to this level are thereafter given more intensive treatment at later steps in the program and will be randomized to more intensive stress-management intervention in a group setting or individually. The primary out-come is subjective distress (intrusion and avoidance) assessed by the Impact of Event Scale (IES). According to the power-analyses, 300 patients are planned to be included in the study and will be followed for one year. Other outcomes are anxiety, depression, quality of life, fatigue, stress in daily living and utilization of hospital services. This will be assessed with well-known psychometric tested questionnaires. Also, the cost-effectiveness of the intervention given in group or individually will be evaluated.

**Discussion:**

This randomized clinical trial will provide additional empirical evidence regarding the effectiveness of a stress-management program given in group or individually during adjuvant therapy in terms of decreased stress, minimizing fatigue, and maintaining or enhancing patients’ quality of life and psychological well-being.

**Trial registration:**

ClinicalTrials.gov Identifier: NCT01555645

## Background

A life threatening illness such as breast cancer can lead to a secondary diagnosis of PTSD (post traumatic stress disorder) [1]. The reported frequency of PTSD for breast cancer patients varies between 2–22%, depending on methods of measurement. At present it is therefore difficult to draw any conclusions about the actual prevalence of PTSD. Intrusive thoughts, avoidance behavior and worry are all common symptoms. Nordin and Glimelius [2] reported that clinical levels of worry or depression in combination with intrusive thoughts in individuals with breast cancer make it possible to identify women who will need psychological support at a later stage of treatment. In a study [3] it was found that measures of avoidance behavior could predict the effects of treatment on an individual’s quality of life. Extensive avoidance behavior at the start of treatment is correlated with deterioration in physical and social functioning as well as impaired general health. Half of the women diagnosed with breast cancer in Sweden are under the age of 65 and at least theoretically gainfully employed [4]. To juggle the demands of work and a family, especially when there are children living at home, is a demanding task [5]. To be diagnosed with breast cancer on top of these demands can be the straw that breaks the camel’s back. At present there is very little research that has examined this combination of stress factors in women diagnosed with breast cancer.

In an international perspective there is a fair amount of evidence which indicates that methods which are based on cognitive behavior therapy can improve health-related quality of life (HRQoL), reduce psychosocial stress and increase perceived Personal control of treatment side-effects and disease symptoms for cancer patients [6,7]. According to SBU (The Swedish Council on Technology Assessment in Health Care) providing cognitive behavior therapy 1–3 months after a traumatic event reduces the occurrence of PTSD in states of intensive stress [8]. At present there is a lack of solid research findings for comparisons of cost-effectiveness and outcome [8].

The research group “Support project” has studied the effect of individual counselling conducted by a specially trained nurse [9]. This intervention was compared to the results of therapy conducted by a psychologist and to a group that received treatment as usual. The project started in 1997 with special training for nurses [10]. The project continued during for the next 5 years and data collection was concluded in January 2002. Patients in this study had been diagnosed with breast cancer and were offered adjuvant treatment. A total of 179 patients were consecutively included. The results showed that both active treatments had a positive effect, measured in terms of increased HRQoL and decreased symptoms of stress (e.g. intrusive thoughts) in comparison to the control group who received treatment as usual [9]. More patients in the control group took advantage of the psychosocial support that was available for all patients in standard care as compared to both intervention groups. The level of satisfaction was generally high in both intervention groups [11]. However patients in the nurse-counselor groups reported that greater satisfaction with interventions for worry, information about their illness, prognosis, tests, treatment as well as general contact with health care facilities than patients in the psychologist groups. Utilization of health care and number of days on sick leave can be attributed to the adjuvant treatment [12]. Both the intervention groups had lower total costs for medical care than the control group who required more days for in-patient care. The conclusions that can be drawn from this study are that the psychosocial interventions were useful for breast cancer patients, that they were relatively inexpensive and that interventions offered by nurses were as effective as those given by psychologists. The results of the “Support Project” described above generated additional questions. Most patients reported relatively high levels of HRQoL as well as relatively low levels of symptoms and side-effects of their illness and treatment. Despite these findings 75% of the patients reported that they had problems for which the psychosocial interventions provided help. A more detailed analysis of the data showed that 80% of the patients in the study reported a high level of stress symptoms initially, such as intrusive thoughts and avoidance behavior. These symptoms remained high throughout the study. Other symptoms such as worry and depression were also reported at high levels initially but these symptoms had decreased to normal levels after 3 months. This indicates that here is a need for closer examination of other factors for breast cancer patients, such as stress-related symptoms and behavior, predictors for stress, effects on role and social functions as well as utilization of heath care, sick leave and return to work settings. In addition there is a need to study how psychosocial interventions with a focus on stress management can influence the above-mentioned factors.

This paper presents the design of a randomized multi-centre trial to evaluate the effectiveness and cost-effectiveness of a stress management intervention using a stepped- care approach. The first step is a low intensity intervention that is given to all patients. Patients who do not respond to this level are thereafter given more intensive treatment at step 2 in the program. They will in step 2 be randomised to more intensive stress-management intervention in a group setting or individual. The hypothesis is that half of the individuals assigned to a low intensity intervention will be significantly improved after treatment. For individuals who continue to have symptoms after low intensity treatment it is hypothesized that continued treatment in a group setting with high intensity interventions will be more cost-effective. In addition the assumption is that reduction of stress symptoms in women with breast cancer will lead to a reduction in socio-economic costs.

## Methods

### Patients

Female patients over the age of 18, with a recent diagnosis of breast cancer and scheduled for adjuvant treatment in the form of chemotherapy, radiation therapy and/or hormonal therapy are eligible for this study and will consecutively be included in the project. Patients will be recruited from three hospitals in the central part of Sweden (Uppsala, Gävle and Falun). Criteria for exclusion are an ongoing psychiatric condition or language deficiencies in Swedish. According to the power calculations, a total sample of 300 patients is necessary.

### Design

**Figure 1:**
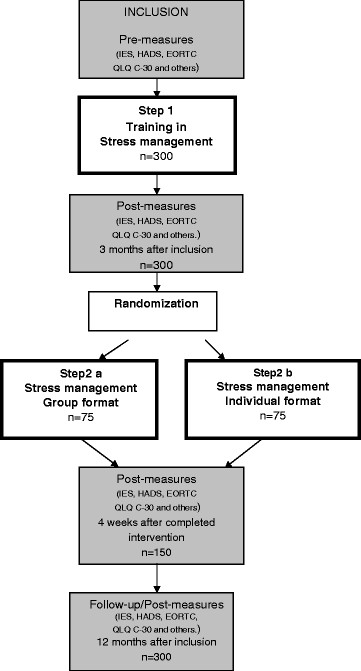
Study design.

The study is a prospective longitudinal intervention study with a stepped-care approach [13], where patients will be randomised to one of two interventions in the final stage of treatment. One of the key concepts in the stepped-care model is that the needs of the individual are matched to the appropriate level of care [13]. The first step is a low intensity intervention that is given to all patients. Patients who do not respond to this level are thereafter given more intensive treatment at later steps in the program. Since methods from CBT (cognitive behavior therapy) have already been shown not only to reduce the risk for PTSD [8] but also to have a positive effect on quality of life and psychological well being for patients with breast cancer [9,14], there is no control group in the study. All patients (n = 300) will receive the first level of the intervention program. All patients (estimated to n = 150) who continue to report stress symptoms after the first step of the intervention program will be given the chance to participate in the second step of the program. The second step includes randomisation to one of two treatments: a stress management group or individual stress management (See Figure 1). With this design it will be possible to study the prevalence of stress related symptoms as well as to study which factors predict which patients will be Clinically Significant Improved (CSI) with a basic level of education about stress management. In addition the study will help to identify those patients who need additional help and to compare the effects of individual and group interventions.

### Intervention

All patients start at the first step of the intervention program and receive detailed information about different stress symptoms and different ways to deal with them. Patients will be given the opportunity to ask questions and will receive written material to be read at home. All patients are evaluated at the end of the intervention (Table 1) to see if they are clinically significant improved (CSI). CSI is defined as a decrease in stress related symptoms as measured by Impact of Event Scale (IES) and/or the Hospital and Depression Scale (HADS) from clinical levels to normal results. Patients who are evaluated as CSI as well as patients who did not report clinical levels on IES or HADS at the start of the intervention leave the program but are followed up during the coming year (Table 1). The remaining patients will be asked if they wish to continue in the program to the next step. Those patients who wish to continue will be randomised to group (Step 2a) or individual stress management (Step 2b). Within a week after randomisation patients will be contacted by telephone to receive information about which group they are assigned to and practical details about the start of the intervention.

**Table 1:** Summary of interventions and measures used at repeated points in time

		***Time of assessment***				
***Questionnaire***	**Inclusion**	*Intervention* *Step 1*	**3 months after inclusion**	*Interventions* *Step 2*	**1 month after completed intervention**	**12 months after completed intervention**
*n*	*300*	*300*	*300*	*150*	*150*	*300*
IES [15]	X		X		X	X
HADS [16]	X		X		X	X
EORTC QLQ C-30 [17]	X		X		X	X
EORTC QLQ BR-23 [18]	X		X		X	X
EQ-5D [19]	X		X		X	X
MFI [20,21]	X		X		X	X
Stress in daily living [22]	X		X		X	X
Self-reported sick leave and use of other professional services	X		X		X	X
Background data	X					X
Patient satisfaction with interventions [11]			X		X	X
PGPQ [23] (n = 150)			X		X	X

### Intervention, Step 2

#### Step 2a

Stress management, group format. Participants (6–8 patients/group) will meet for 2 hours every week for a total of 20 hours. In the intervals between the group meetings patients will be asked to do homework. This homework can entail practicing problem-solving techniques, keeping a diary, practicing relaxation or participating in physical activities. Each group meeting has a specific subject, derived from the 6 components in the intervention [24]. These components concern 1) basic knowledge about cancer, treatment, healthy living and stress reactions, 2) self-awareness with the help of a diary for thoughts, feelings and behavior, as well as group discussions of specific cases 3) instruction in various techniques about how to express negative feelings, how to communicate with others more effectively how to change behaviors related to stress, anger, worry and depression 4) training these techniques in real-life situations outside the group, 5) cognitive restructuring with the help of diaries, techniques and group dynamics and 6) spirituality, in a broad sense, with group discussions of quality of life, values, self-confidence and optimism. Written materials, case studies and video presentations will be used.

### Step 2b Individual intervention

The methods and techniques will be the same as those used in the group intervention. The first session will be used for a detailed assessment of the individual’s psychosocial problems, as used in earlier studies [9,11]. The sessions will last 45–60 minutes. The number of sessions will depend on the individual patient’s problems and the joint assessment made by the patient and nurse together. The total number of sessions will be at least 4, with a maximum of 8. The contents of the sessions are Session 1: Assessment, Session 2: Analysis of diary (self-registration) and suggestions for problem management, Session 3: Evaluation of problem management skills Session 4: Follow-up and conclusion of the intervention. When necessary Sessions 5–8 will address specific obstacles and continued practice.

### Implementation

Patients will receive written information about the project when they receive notice for a doctor’s appointment to discuss adjuvant treatment for breast cancer. They will be asked if they wish to participate in the project for stress management by their doctor or by a member of the project group after the appointment. If they answer “yes” they will then receive the first set of questionnaires. Their answers constitute the baseline measures as well as a prevalence screening for stress symptoms. When the questionnaires are completed the patient will receive a written invitation to Step 1 of the intervention program, i.e. to a lecture on stress management. The intervention program will take place in Falun, Gävle or Uppsala, and be conducted by specially trained nurses who will receive continual supervision. The nurses who conduct the intervention program will be trained in general stress management, psychosocial oncology as well as assessment and treatment of psychosocial problems.

### Data collection

Data will be collected by use of well-known questionnaires. An outline of the assessment points and questionnaires are included in Table 1. When patients are included in the study, they were mailed the baseline questionnaires, which included a signed consent to participate and a prepaid envelope. Later measurements will be sent out to the patients’ homes, together with a stamped, pre-addressed envelope. Patients who have not returned the completed questionnaires after 14 days will be contacted by telephone by a member of the project staff to insure as high a response rate as possible. Information about the patients’ demographic and medical background will be obtained from their medical records. Utilization of hospital services will be collected from the hospitals’ computer systems. Information about utilization of other professional care facilities will be collected from patients’ self-reports. Sick-leave information will also be collected by self-report. The nurse-counsellor will record attendance at individual and group sessions.

### Data analysis

SPSS® ANOVA will be used to analyze differences between groups and for repeated measures over time for the continuous variables while nominal (categorical) variables will be tested with chi square (exact). Although not all the criteria for normal distribution are met by this selection the parametric tests are robust enough to be used. Patients’ levels of stress related symptoms will be categorized according to recognized cut-off points (See references in Table 1). A hierarchical linear regression analysis will be used to examine which variables predict stress symptoms and utilization of health care services as well as sick leave and return to work. Power calculations have been done for IES based on data from Support Project. On the basis of these conditions (power = 0.8, p = .05 and effect size = 0.59) at least 64 patients must be included in each group (i.e. a total of 128) patients) to detect a significant difference on IES.

### Ethical considerations

The project has been approved the Regional Ethical Review Board in Uppsala 20090327, Diary number 2008/382. A written informed consent for participation in the study was obtained from participants. The interventions in the project will be provided by specially trained staff and supervised continuously so that any potentially harmful interventions can be detected early in treatment and corrected immediately. Filling in numerous questionnaires can be demanding. The questionnaires used in this study have been chosen with care, both in terms of length and number. However, if the number or length of questionnaires were to be further reduced it could make it difficult to reach any conclusions, which would reduce the value of this study for patients and grant-givers alike.

## Discussion

The interventions that are planned for in this project are directed towards symptoms that can make or break the course of illness for an individual patient. Hopefully the project will show that a stepped-care approach can help and support individual patients at the right level of care. All patients will receive stress management training. Those who require more help will receive additional treatment for their symptoms in the form of individual or group counselling. Health care and society may gain substantially from the planned intervention program, both in the form of individualized psychosocial support and reduced utilization of health care, preserved work capacity or a quicker return to work. The more intensive support will only be offered to those patients who show a clear need. For research purposes it is also important to increase what is known about stressors that are specific to women diagnosed with breast cancer and to develop interventions that are directed at these symptoms. This study will be conducted in three different counties in central Sweden which contributes to increased generalization and dissemination of the results of the study. This can lead to implementation of similar evidenced-based psychosocial care for cancer patients within a large geographical area.

Limitations of the study should be noted as well. The lack of a true control-group in step-2 can result in difficulties to interpret the effects of the stress-intervention. If there is a positive result, we cannot be absolutely sure that this effect would not be present in an untreated group. However, we consider not to have a untreated control since methods from CBT (cognitive behavior therapy) have already been shown not only to reduce the risk for PTSD [8] but also to have a positive effect on quality of life and psychological well being for patients with breast cancer [9,14]. It would not be ethical to withdraw patients in need from a treatment that has been found effective.

## Competing interests

The authors declare that they have no competing interests.

## Authors’ contributions

KN, JA, GB, M-LF and CA were involved in the design of the study. KN and CA are the principal investigators of this study. RR and SB is conducting this research in fulfillment of a PhD, and will together with CA be responsible for data collection, and RR, SB and CA will together with KN, JA and M-LF do the analysis and interpretation. All authors have read and approved the final version of the manuscript.

## Pre-publication history

The pre-publication history for this paper can be accessed here:

http://www.biomedcentral.com/1471-2407/12/167/prepub
